# Adaptive cohort design and LAT1 expression scale: study protocol for a Phase 2a trial of QBS72S in breast cancer brain metastases

**DOI:** 10.1186/s12885-025-14282-x

**Published:** 2025-08-15

**Authors:** Rukayat Taiwo, Paul M. Harary, Thy T. H. Trinh, Monica Granucci, Sophie Bertrand, Brandon Carlson-Clarke, Sophia B. Chernikova, Kate Therkelsen, Mili Arora, Michelle E. Melisko, Michael Iv, Hannes Vogel, Summer Han, Christine Xie, Susie Brain, Vivian Lee, Krishna L. Bharani, Seema Nagpal, Melanie Hayden Gephart

**Affiliations:** 1 Department of Neurosurgery, Stanford University School of Medicine, Stanford Cancer Center, 875 Blake Wilbur Dr. Clinic A MC 6560, Stanford, CA 94305 USA; 2 Department of Neurology and Neurological Sciences, Stanford Cancer Institute, Stanford, CA USA; 3 Division of Hematology and Oncology, University of California, Davis Comprehensive Cancer Center, University of California, Davis School of Medicine, Sacramento, CA USA; 4 Division of Hematology/Oncology, Department of Medicine, University of California, San Francisco, San Francisco, CA USA; 5 Department of Radiology, Stanford University School of Medicine, Stanford, CA USA; 6 Department of Pathology, Stanford University School of Medicine, Stanford, CA USA; 7 Quantitative Sciences Unit, Stanford University School of Medicine, Stanford, CA USA; 8 Patient Advocate, Komen Advocates in Science, Stanford, USA

**Keywords:** Breast cancer, Leptomeningeal disease, Chemotherapy, Expression profiling, Liquid biopsy

## Abstract

**Background:**

Breast cancer is the most common cause of cancer death among women and frequently metastasizes to the brain. Up to 30% of patients with breast-to-brain metastases will develop leptomeningeal disease (LMD), with poor survival, rapid neurologic decline, and no durable treatment options. The novel agent QBS72S, also known as QBS10072S, is designed to leverage the high expression of L-type amino acid transporter 1 (LAT1) on breast cancer cells and the blood-brain barrier. By conjugating an amino acid analogue with a DNA alkylating moiety, QBS72S can exploit LAT1 for specific delivery into the brain and metastatic tumor cells.

**Methods:**

We designed a single-arm, Phase 2a study to test the preliminary efficacy and safety of QBS72S for breast-to-brain metastasis in two distinct cohorts: intraparenchymal metastasis (Cohort 1; no LMD), and LMD (Cohort 2; with or without intraparenchymal metastasis). The primary endpoint is overall response rate across evaluable participants in Cohort 1. Secondary endpoints include progression-free survival, overall survival, duration of response, and treatment-related adverse events in Cohort 1. Exploratory endpoints include correlation of LAT1 expression in formalin-fixed paraffin-embedded samples with treatment response, CSF pharmacokinetics, perfusion MRI, and novel CSF-based biomarkers.

**Discussion:**

Adaptive clinical trial design enables rapid enrollment and tailored endpoints for patient cohorts with baseline disparate outcomes. Our LAT1 staining protocol will allow ongoing trials in glioblastoma (NCT02977780) and future studies in brain metastases to correlate LAT1 expression to drug efficacy. Our exploratory endpoints may facilitate identification of more rapid and reliable biomarkers of LMD treatment response and resistance.

**Trial registration:**

ClinicalTrials.gov NCT05305365.

**Supplementary Information:**

The online version contains supplementary material available at 10.1186/s12885-025-14282-x.

## Background

Brain metastases (BrM) are the most common type of brain tumor, with an estimated incidence of up to 300,000 new cases diagnosed each year [1]. BrM are particularly challenging to treat because the blood-brain barrier impedes entry of the vast majority of systemic therapeutic agents. In women, breast cancer is the second most common cause of cancer death, and up to 40% of women with advanced breast cancer will develop BrM [2–4]. Breast-to-brain metastases impart short survival times (6–9 months from BrM diagnosis) and a significant decrease in quality of life due to BrM treatment sequelae [5, 6]. Limited systemic therapeutic options exist with sufficient brain permeability, and those available tend to target specific receptor subtypes (e.g., HER2) [7]. Although some patients have isolated BrM, many have parallel progression of their systemic disease which would not be alleviated by neurosurgery, brain radiation, or intrathecal therapy. This highlights the need for improved systemic therapy with high central nervous system (CNS) penetration.

Breast-to-brain metastases remain the most common etiology of leptomeningeal disease (LMD) [8], in which cancer cells disseminate on the surface of the brain and spinal cord, along exiting nerves, and within the cerebrospinal fluid (CSF). LMD diagnosis portends a dismal prognosis with acute decline in neurologic function, quality of life, and survival [9, 10]. LMD’s diffuse nature requires systemic therapy or craniospinal irradiation (CSI); yet current FDA-approved drugs have limited efficacy in LMD, and many patients cannot tolerate the toxicity and neurologic sequelae of CSI [11]. Likewise, given the insensitive nature of MRI and cytology, biomarkers to track LMD response and resistance to therapy are critically needed.

The L-type amino acid transporter 1 (LAT1) is normally expressed on the endothelial cells that form the blood–brain barrier, where it facilitates the transport of neutral branched chain and aromatic amino acids [12–14]. LAT1 has been leveraged pharmacologically for entry into the brain by commonly known amino acid-based drugs, such as L-Dopa for Parkinson’s disease and gabapentin for seizures [15, 16]. Metastatic cancers, and particularly BrM, express high levels of LAT1 [17]; triple negative breast cancer (TNBC), has high levels of LAT1 expression [18], high risk of BrM including LMD, and generally lacks targeted treatment options [18].

QBS72S, also known as QBS10072S [18], is a novel amino acid analog integrated with a DNA alkylating moiety that leverages LAT1 for active transport into the brain, and cancer targeting due to elevated LAT1 expression in metastatic cells. Our preclinical study shows that QBS72S selectively kills TNBC cells in vitro, and effectively decreases tumor growth and prolongs survival in vivo in an aggressive human TNBC BrM mouse model that includes LMD [18]. Given elevated LAT1 expression on metastatic cancer in general (i.e., not necessarily specific to BrM), if QBS72S were to prove efficacious in BrM, it should be likewise active against the patient’s systemic disease.

We developed an adaptive, single-arm, Phase 2a study to test the preliminary efficacy and safety of the cytotoxic agent QBS72S for the treatment of breast-to-brain metastasis and separated patients in two distinct cohorts: intraparenchymal metastasis (Cohort 1; no LMD), and LMD (Cohort 2; with or without intraparenchymal metastasis). Since patients with BrM and LMD do not routinely undergo CNS biopsy, we developed a novel LAT1 staining protocol and grading scale used post hoc, to test any available patient sample and correlate with treatment response, we developed a novel LAT1 staining protocol and grading scale. Finally, given the challenges of diagnosis and monitoring of LMD, we developed exploratory endpoints to identify more rapid and reliable biomarkers of treatment response and resistance, to help increase throughput of future LMD-focused clinical trials.

## Methods/design

### Study design

**Fig. 1:**
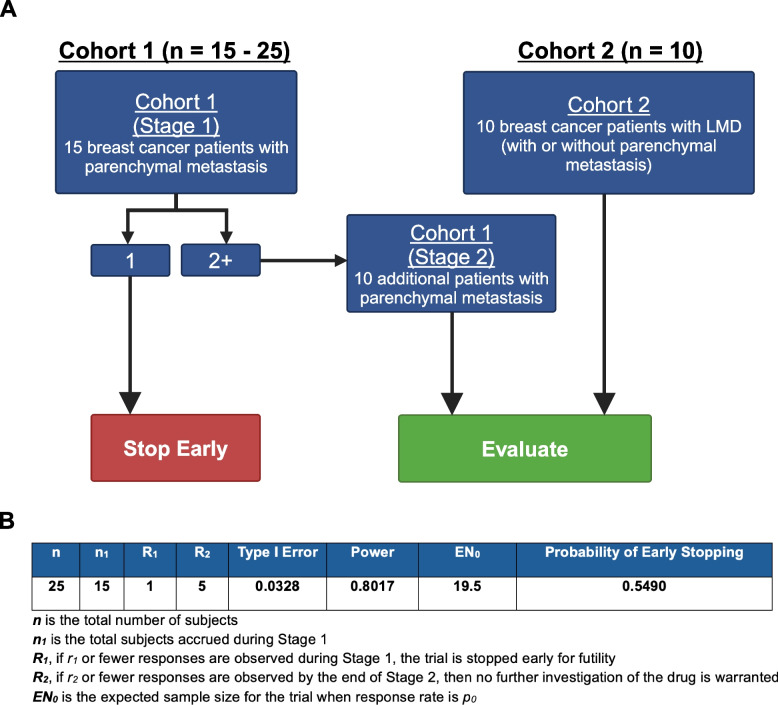
Protocol schema. Cohort 1 was designed for patients with breast-to-brain intraparenchymal metastases and no LMD. If at least 2 of the first 15 evaluable patients met the primary endpoint (Overall Response Rate) in Cohort 1, then an additional 10 patients would be included and proceed to the second stage. To allow for the inclusion of patients with LMD, Cohort 2 was created to include patients with breast-to-brain LMD (with or without intraparenchymal metastases)

We designed a prospective, open-label, non-randomized, Phase 2a clinical trial at Stanford Health Care investigating the use of QBS72S in patients with either intraparenchymal metastases (Cohort 1, no LMD) or LMD (Cohort 2, ± intraparenchymal metastases). The intervention model of this study was single group assignment and employs a Simon’s two stage design [19] (Fig. 1). This protocol was approved by the Stanford Institutional Review Board (IRB no. 63041), and all patients provided written informed consent.

The first stage of Cohort 1 consists of 15 patients with at least one untreated brain metastasis ≥ 3 mm and no radiographic evidence of LMD. Given challenges with disease monitoring (insensitive cytology and MRI), rapid neurologic symptom progression, and severely limited survival in patients with breast cancer LMD, we created Cohort 2 to allow for the inclusion of patients with progressive LMD, an underserved population generally excluded from clinical trials. Cohort 2 allows 10 participants with breast cancer LMD (with or without intraparenchymal brain metastases). In both groups, evaluable participants are defined as having received at least one complete dose of QBS72S at 18 mg/m^2^, and one additional MRI scan at least one month following dosing. If the patient does not show treatment response and LAT1 Expression Score = 0 (see below; no target expression) in a metastatic cancer FFPE specimen, then the patient can be replaced post hoc with an additional patient. The primary endpoint of tumor response is based on Cohort 1 alone; if one or fewer responses in the 15 evaluable participants is observed, the study will be stopped. If there are two or more responses, then 10 evaluable additional participants will be accrued for the second stage to form Cohort 1 Stage 2 for a total of 25 evaluable participants in Cohort 1.

### Study objectives

The primary endpoint of this trial is to determine the preliminary intracranial anti-tumor activity of QBS72S through the overall response rate (ORR) across evaluable participants in Cohort 1. Secondary endpoints include measurement of progression free survival (PFS), overall survival (OS), durations of response (DoR), and a safety event listing from all Cohort 1 participants, regardless of primary objective evaluability. Exploratory endpoints include: i) PFS, OS, and durations of CNS response (Cohort 2); ii) ORR, PFS, DoR of systemic disease; iii) the relationship between LAT1 expression and response to treatment; and iv) MRI perfusion metrics, CSF pharmacokinetics, and CSF biomarker development. Upon completion of the trial, patients will continue with the current standard of care, including radiation. For many patients with LMD, this trial represents a final treatment option prior to consideration of WBRT, CSI, or palliative care only. Patients with treatment response are permitted to continue on trial provided they maintain a favorable functional status, experience limited treatment toxicity, and show no signs of disease progression.

### Enrollment prediction

Although the study will be open to any patient with breast-to-brain metastases, patients with BrM tend to have advanced disease, extensive treatment history, and with LMD, neurologic symptom burden, which would limit travel. As such, we created a collaborative recruitment strategy including potential participant streams from Stanford, UCSF, and UC Davis, combined with active communication with the patients’ local medical oncology teams. Researchers consulted Stanford Medicine’s database of archival patient visits to Stanford Cancer Center’s Neuro-Oncology and Neuro-Radiology clinics for incidence of potentially eligible participants from 2008 to 2018, then added UCSF’s Breast Oncology Division’s potential participant estimates. This combined tally was then lowered to account for 50% of potentially eligible participants choosing to pursue other options.

### Informed consent and safety

The Stanford Institutional Review Board (IRB) approved the study protocol. All study personnel qualified to conduct the informed consent process completed Human Subjects Protection training. Written informed consent will be required prior to the conduct of any study-specific procedures in accordance with institutional policies. The participant may withdraw consent and discontinue participation in the study at any time without negative impact on further treatment. For eligible participants with a disease condition limiting capacity to provide full informed consent, consent may be obtained by Legally Authorized Representatives (LAR) upon verification of appropriate documentation permitting LAR to act on behalf of the participant. All eligible participants who provide informed consent will be registered, assigned a unique participant identification number, and stored in HIPAA-compliant eCRF database (REDCap) on an encrypted, password protected computer. The Principal Investigator (PI) and the Stanford Cancer Institute (SCI) Data Safety Monitoring Committee (DSMC) will conduct data and safety monitoring activities.

### Patient eligibility criteria

Adult patients with untreated or progressive intraparenchymal or LMD BrM will be referred and pre-screened for enrollment (Table 1). Specifically, Cohorts 1 and 2 will both be comprised of participants having previously received at least one cytotoxic chemotherapy regimen with 1) histologically confirmed breast cancer primary or metastatic tumor, and 2) intraparenchymal or LMD BrM. Patients will be placed in Cohort 1 if intraparenchymal tumors > 3 mm^2^ are identified on gadolinium contrast weighted MRI scan with no LMD. Patients will be placed in Cohort 2 if they have a diagnosis of LMD (diagnosed by MRI or cytology), with or without the presence of intraparenchymal BrM. All patients must have a Karnofsky Performance Status (KPS) of ≥ 60. Washout criteria include 28 days from radiation or any major surgery, variable time from systemic therapy (Supplemental Table 1), and 1 week from minor procedures (including the placement of a ventricular access device – VAD, or ventriculoperitoneal shunt). Exclusion criteria include insufficient blood cell counts (e.g., platelet < 100), other active malignancy within 3 years prior to enrollment (except for adequately treated basal cell or squamous cell skin cancer or carcinoma in situ). All Cohort 2 LMD patients are required to have a VAD. For detailed inclusion and exclusion criteria, refer to Supplemental Table 1. Patients can elect for a pre-screening video visit and consultation with the Clinical Research Coordinator to help ensure eligibility, reduce travel burdens, and streamline enrollment.

Brain metastases are not generally biopsied prior to treatment, and no CSF test exists to determine the gene expression profile of LMD. In addition, given the fragmented nature of our patients’ care, tissue source variety (e.g., primary breast, lymph node, other metastatic site) and scarcity/exhaustion, potentially long interval between primary or metastatic cancer diagnosis and the development of BrM and LMD, rapid BrM disease progression, insufficient data on the direct relationship between LAT1 expression and QBS72S efficacy, and the lack of a standardized LAT1 staining protocol, we do not require confirmation of LAT1 expression prior to trial enrollment. While evidence of LAT1 expression is not required for enrollment in the trial, patients who are later found to have no LAT1 expression (Score = 0, see below) in available tissue will not be considered evaluable. Accordingly, our statistical model allows for replacement of subjects with no LAT1 expression with an evaluable patient.

**Table 1:** Schedule of treatments and assessments. Screening would occur within 28 days prior to Cycle 1 Day 1 (C1D1). The end-of-treatment (EOT) visit would occur within 7 days of discontinuation of study treatment. QBS72S would be administered IV once every 28 (± 3) days. Systemic imaging would be performed once every 6 weeks (+ systemic disease) or 3 months (no systemic disease). All participants who discontinued study treatment would have a safety follow-up visit approximately 30 (±7) days after EOT to collect any subsequent adverse events

Study Phase	Screening (Days)	Treatment								End of Treatment	Safety FollowUp Visit	Long Term Follow-Up
Day of Phase	**−28 to −1**	**Cycle 1**				**Cycle 2**		**Cycle 3+**				
		**D1**	**D8**	**D15**	**D22**	**D1**	**D15**	**D1**	**D15**			
Allowed Time Window		**± 0**	**± 2**	**± 2**	**± 2**	**± 2**	**± 2**	**± 3**	**± 3**			
Study Day	**−28 to −1**	**1**	**8**	**15**	**22**	**29**	**36**					
QBS72S infusion		**x**				**x**		**x**				
Place Ommaya (LMD only)	**x (−28 to −7)**											
Pregnancy testing	**x (−7 to −1)**	**x**				**x**		**x**		**x**		
Contraception check	**x**											
MRI	**x (−14 to −1)**	**x**	*-------------------* ***q6wk per Standard of Care*** *-------------------*							**x**		**x**
Systemic imaging		**x**	* --------* ***q6wk or q3mo, based on presence of systemic disease on screening*** *--------*							**x**		**x**
Pharmacokinetics (LMD only)		2x*										
Biomarker sample (LMD only)		2x*	**x**	**x**	**x**	**x**	**x**	**x**	**x**	**x**		**x**
Archival tumor biopsy	**x**											
Adverse events		**x**								**x**	**x**	
Survival follow-up												**(q8wk ±21d)**

^*^Sample obtained before and after drug administration

### Treatment

QBS72S will be administered IV as a single dose of 18 mg/m^2^ to all enrolled participants in Cohorts 1 and 2, every 28 days ± 3 days. This dose and schedule is based on the Phase 1 clinical trial Cohort Review Committee [20]. In subsequent cycles, participants can be dose reduced to 12 mg/m^2^ if needed, or the full dose delayed by up to 28 calendar days, to allow for events such as palliative radiation, minor surgical intervention, or drug-related toxicity (e.g., bone marrow suppression). All participants can receive infusions of QBS72S until confirmed disease progression, unacceptable toxicity, participant preference, or the medical team’s discretion acting in the patient’s best interest. If progressive disease occurs, continuation of study drug will be allowable if the multidisciplinary medical team finds evidence of clinical benefit, absence of any serious toxicity, participant preference, and after discussion with the PI. It should be noted that patients in Cohort 2 (LMD) frequently will have exhausted all viable treatment options; given the likely transition to palliative treatment only, end of treatment visits have limited assessments and can be performed over video visit to reduce patient hardship.

### Outcome assessments

The primary endpoint of overall response rate (ORR) across evaluable participants in Cohort 1 is based on the modified Response Assessment in Neuro‑Oncology for Brain Metastases (mRANO-BM) and RECIST 1.1 response criteria [20, 21]. MRI tumor measurements are obtained at Stanford’s MRI facilities by board certified neuroradiologists using Stanford’s standard of care brain tumor scan protocols. This protocol at a minimum includes the following sequences: T1-weighted images (T1WI) with and without contrast, T2-weighted images (T2WI), Fluid Attenuated Inversion Recovery (FLAIR) images as well as perfusion imaging. Participants have a minimum of one to a maximum of five target lesions ≥ 3 mm indexed and measured for baseline maximum diameter. The number of target lesions may increase during data collection if new lesions measuring ≥ 3 mm develop while the participant is on study. Percentage growth or reduction in sum total diameter of target lesions is measured by comparing baseline MRI tumor measurements to MRI tumor measurements taken at each timepoint following C1D1 until the participant was removed from study. A successful response is defined as ≥ 30% decrease in sum total diameter of all target lesions compared with baseline across two or more sets of scans, provided that steroid dose is lower or the same as baseline, and neurological evaluations were stable or improved. Response is broken down into the following categories: complete response, partial response, progressive disease, and stable disease. Both complete and partial response qualify as responses for the purposes of this study (Table 2). Patients with stable disease are categorized with responding patients as garnering CNS benefit.

**Table 2:** Overall response rate criteria for primary objective evaluable study participants (Cohort 1). Participants would have one to five target lesions (≥3mm) measured for baseline maximum diameter

State of disease	Change in maximum diameter of lesion
Preliminary complete response	100% decrease
Confirmed complete response	100% decrease for 4 + weeks
Preliminary partial response	≥ 30% decrease
Confirmed partial response	≥ 30% decrease for 4 + weeks
Preliminary progressive disease	≥ 25% increase
Confirmed progressive disease	≥ 25% increase for 6 + weeks
Stable disease	Any response not complete, partial, or progressive
Pseudoresponse	Any preliminary complete or preliminary partial response that does not evolve into confirmed complete response or confirmed partial response on the following scan
Pseudoprogression	Any preliminary progressive disease that does not evolve into confirmed progressive disease on the following scan

The interim ORR will be evaluated for the first stage after the 15th Cohort 1 participant completed treatment and tumor growth was assessed; if there are < 2 responses the study will be stopped. The ORR for the full study will be determined after the 25th participant completes treatment and tumor growth is assessed. This stopping rule was set to ensure the given study design provides 80% power and > 5% type I error rate (Fig. 1). We will summarize ORR for all participants with a binomial response rate. The Clopper-Pearson method [22] will be used to estimate the two-sided 95% CI. According to Simon’s two-stage design, the null hypothesis will be rejected if six or more responses are observed in 25 evaluable participants (Fig. 1).

Secondary objectives include PFS, OS, DoR for all Cohort 1 participants, and will be estimated using Kaplan–Meier techniques [23, 24]; time from diagnosis or C1D1 to event distribution for PFS, OS, and DoR Median survival time along with a 95% CI will be constructed [25, 26]. Rates at fixed time points will be derived from the Kaplan Meier estimate and corresponding confidence interval will be derived based on the Greenwood formula [27]. For both Cohorts, PFS is defined as the length of time that participants did not experience confirmed disease progression measured from the time of study enrollment until disease progression is observed, or the participant passes away. Confirmed progressive disease will be determined by either the PI or one of the research physicians. OS will be measured from the date of diagnosis of (progressive) study disease until the participant passes away. Durations of response (DoR) is divided into two: DoR_prog_ and DoR_DOD_. DoR_prog_ is to be measured from first dose of QBS72S until disease progression; DoR_DOD_ is to be measured from first dose of QBS72S until the participant passes away. Both BrM and systemic disease progression will be recorded.

Safety is defined as a listing of all adverse events, graded according to CTCAE v5.0 [28], and all events will be recorded. The safety analysis set will include all participants who are enrolled into the study and who received the planned Cycle 1 dose of QBS72S, and either were assessed at both of the Cycle 1 safety assessments (Days 14 and 28) or withdrew due to treatment related toxicity. All baseline characteristics, safety, immunogenicity, and efficacy analyses will be conducted based on the safety analysis set.

**Fig. 2:**
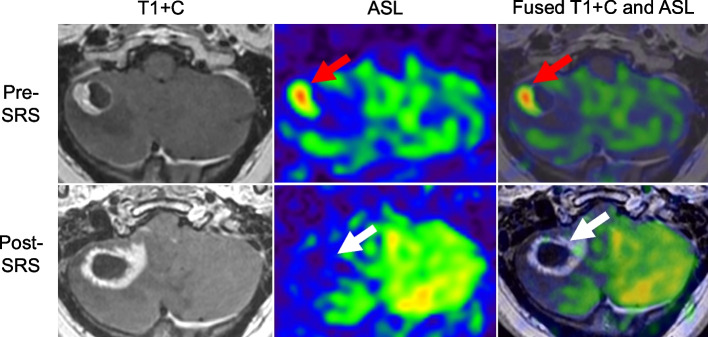
*Top row*: A solid and cystic metastasis in the right cerebellum with high blood flow on ASL perfusion and fused post-contrast T1 and ASL images (*red arrows*). *Bottom row*: Enlargement of the cystic lesion with thicker but irregular rind of enhancement, 13 months after initial stereotactic radiosurgery. No elevated blood flow is seen on ASL perfusion and fused post-contrast T1 and ASL images (*white arrows*). Surgical resection of the suspected metastasis performed immediately after the post-stereotactic radiosurgery MRI revealed extensive radiation necrosis and absence of tumor

VADs are standard of care in patients with LMD to allow for CSF access; VAD is an inclusion criterium for Cohort 2. Patients in Cohort 2 will undergo CSF access at each clinic visit. On C1D1, patients will have CSF access prior to and after QBS dosing, to allow for exploratory CSF pharmacokinetic (PK) specimen analysis. CSF bioavailability of QBS72S will be measured by CSF mass spectrometry, and compared to serum levels, with results expressed as the CSF to plasma drug ratio [29]. Subsequent CSF access will be sent for exploratory development of CSF biomarkers, including comprehensive cellular and cell free gene and protein expression analyses [30]. Additional exploratory endpoints will include MRI perfusion imaging to differentiate between parenchymal lesion enlargement and radiation necrosis **(**Fig. 2**)**. LAT1 expression profile of all available tissue (see below) will be determined and correlated with patient treatment response and molecular biomarkers.

### LAT1 expression scale development

To explore the relationship between LAT1 expression and response to treatment, we developed an immunohistochemical (IHC) based analysis of FFPE archival tumor specimens, using a monoclonal antibody against LAT1/SLC7A5. In brief, sections are cut from FFPE tissue blocks at a thickness of 4 μm sections and incubated with anti-LAT1/SLC7A5 antibody (1:500, clone EPR17573, Abcam ab208776). A biotinylated secondary antibody is then applied, followed by avidin–biotin-peroxidase complex (VectorLabs, PK-6101). Staining signal is further developed using DAB substrate (3,3’-diaminobenzidine) (VectorLabs, SK-4100).

**Fig. 3:**
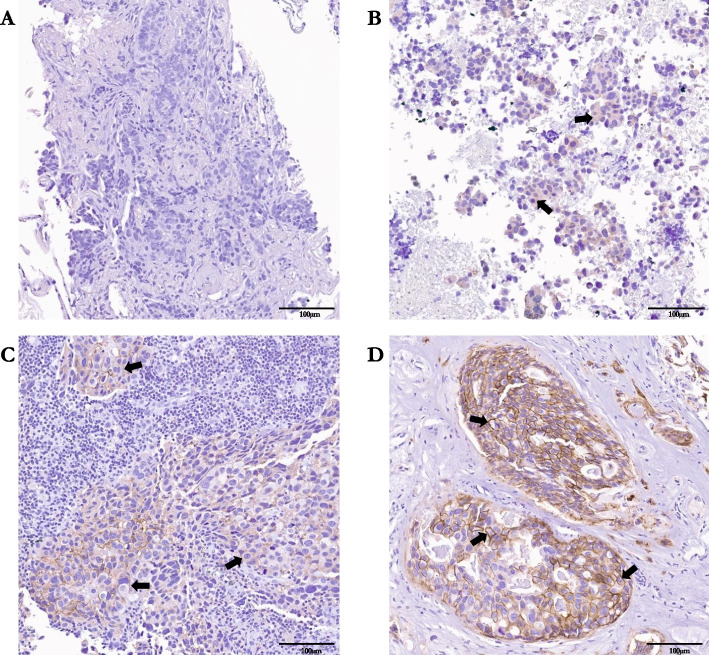
Immunohistochemical quantification of LAT1 expression at different metastatic sites in patients with primary breast cancer. IHC was performed on sections from FFPE slides of metastatic breast cancer tumors to determine LAT1 expression. LAT1 staining was evaluated for signal intensity and location (cytoplasm vs membrane) to create a composite score from 0–3. Representative scores of 0 = negative, 1 = cytoplasmic stain or weak overall stain, 2 = medium membranous stain, 3 = strong membranous stain are represented by panels **A** – **D**, respectively. **A** Upper lung mass. **B** Cervix, **C** Lymph node, **D** Brain tumor. Arrow heads identify regions of positive staining

To generate the initial LAT1 grading scale, we tested LAT1 expression in 13 patients with breast cancer metastases sampled from the lung, cervical lymph node, eye muscle, pleural fluid, uterus, ovaries, cervix, axilla, spine, liver, bone marrow, or brain. Using a four-tiered qualitative grading scale, LAT1 staining was evaluated for signal intensity and location (cytoplasm vs membrane) [31] to create a composite score of 0 = negative, 1 = cytoplasmic stain or weak overall stain, 2 = medium membranous stain, 3 = strong membranous stain (Fig. 3). The composite score depended upon the most prevalent positive staining pattern of the tumor on the tissue. Tissue was considered LAT1-positive if ≥ 10% of the tumor demonstrated a staining pattern of 2 or 3; otherwise, it was classified as negative. All IHC analysis was independently verified by a board-certified neuropathologist. For the trial, any available tissue will be obtained and tested, including primary tumor, lymph node, non-CNS metastatic site (e.g. bone, liver, lung, pleural fluid), and brain metastasis.

## Discussion

### Current options for brain metastasis treatment are insufficient

Current FDA-approved options for treating breast cancer BrM, especially TNBC and LMD, are severely limited (Table 3). In patients with breast cancer, current systemic therapies with CNS penetration mainly target the human epidermal growth factor receptor (HER2), such as tyrosine kinase inhibitors (e.g., laptinib, neratinib, and tucatinib), monoclonal antibodies (e.g., trastuzumab), and antibody–drug conjugates (e.g., trastuzumab-emtansine, and trastuzumab-deruxtecan). Although a combination of tucatinib, trastuzumab, and capecitabine improved PFS in patients with HER2-positive BrM [32], median PFS was only 7 months and patients with LMD were excluded. Hormone receptor-positive BrM may respond to endocrine therapies such as tamoxifen, letrozole, and cyclin dependent kinase inhibitors like abemaciclib [33]. Unfortunately, most patients with BrM will have already progressed on hormone or HER2 targeted therapy. TNBC lack specific targets, limiting options mainly to chemotherapeutic agents with blood brain barrier permeability like capecitabine, platinum compounds, and temozolomide, though efficacy is limited, particularly in LMD [34]. Ongoing Phase 2 trials are exploring bevacizumab, an antibody against vascular endothelial growth factor receptor (VEGF) in combination with other cytotoxic chemotherapy agents such as etoposide and cisplatin, or carboplatin [35]. Treatment and trials for breast cancer LMD are even more scarce, limited mostly to case series and a small number of early phase clinical trials [36, 37].

**Table 3:** Current treatment options for breast cancer brain metastases. Many systemic therapies have poor blood-brain barrier penetrance, limiting treatment options for brain metastases [37, 41]. C This challenge is magnified in TNBC and LMD where approved therapies are especially limited [42]

Treatment modality	Treatment
**Systemic therapy**	
HER2-positive	*Tyrosine kinase inhibitors:* • Laptinib • Neratinib • Tucatinib *Monoclonal antibodies:* • Trastuzumab *Antibody–drug conjugates:* • Trastuzumab-emtansine • Trastuzumab-deruxtecan *Adjuvant chemotherapies:* • Capecitabine
Hormone receptor-positive	• Endocrine-based agents (fulvestrant, tamoxifen, aromatase inhibitors) • CDK inhibition (abemaciclib) • PI3K inhibition (alpelisib) • PARP1 inihibition (talazoparib), if *BRCA1/2* mutation-positive • Chemotherapies (capecitabine, eribulin, carboplatin)
TNBC	• PARP1 (talazoparib), if *BRCA1/2* mutation-positive • Chemotherapies (paclitaxel, eribulin)
**Local therapy**	
Surgical resection	• Surgery • Laser interstitial thermal therapy
Radiotherapy	• Whole-brain radiotherapy • Stereotactic radiotherapy

### Novel targeting of LAT1 in brain metastases by QBS72S

QBS72S represents a novel approach to BrM treatment due to its targeting of DNA repair defects and leveraging LAT1-mediated transport into the brain [18]. These features may enable increased tumor cytotoxicity while mitigating systemic and neuro toxicity. An amino acid moiety within QBS72S facilitates transport across the blood–brain barrier as well as targeting of LAT1-overexpressing cells. In addition, QBS72S contains a nitrogen mustard group that induces DNA inter-strand crosslinking [38, 39], resulting in increased sensitivity of cells with reduced DNA repair to the compound. Importantly, TNBCs are known to have DNA repair defects [40–43], with more than 70% of cases being affected by a mutation in homologous repair [42, 43]. This bifunctionality provides the basis for the therapeutic window of QBS72S, potentially allowing for preferential killing of breast cancer cells while sparing healthy tissue. Investigation of QBS27S in vitro using a human TNBC cell line and a brain-tropic variant cell line demonstrated reduced cell growth following drug treatment [18]. Furthermore, in a rodent model of TNBC CNS metastasis, systemic administration of the drug resulted in increased animal survival and delayed tumor progression [18]. Due to these characteristics, QBS72S has potential as a single-agent treatment for both solid parenchymal BrM and diffuse LMD.

### Adaptive clinical trial design and implementation for progressive brain metastases and LMD

Clinical trials for BrM face numerous design, implementation, and interpretation challenges (Table 4), which we have explicitly attempted to overcome. For example, BrM clinical trials encounter challenges in tracking response to therapy, disparate outcomes for patients with intraparenchymal vs leptomeningeal disease, confirmation of target expression, and speed of enrollment given rapid clinical progression. Alisha et al. conducted a systematic review in 2023 examining the proportion of Phase 3 clinical trials for patients with metastatic breast cancer, lung cancer, and melanoma that included patients with LMD [44]. Of the 244 Phase 3 randomized clinical trials examined, 27.5% explicitly excluded LMD patients, 69.3% did not specify LMD in inclusion/exclusion criteria, and 3.3% allowed enrollment of LMD patients under the condition that they be asymptomatic or previously treated; none of the breast primary trials included patients with LMD [45–47]. We designed our trial for rapid identification and enrollment of patients with BrM and LMD, with statistical allowances for post hoc LAT1 expression testing.

**Table 4:** Common challenges in clinical trials for brain metastases. Our adaptive trial design for patients with brain metastases and LMD seeks to overcome these challenges

Challenge	Potential solution
Differentiating pseudoprogression from true progression on follow-up imaging	• Inclusion of a “preliminary disease progression” category, with consecutive scans showing sustained increase in lesion diameter required for confirmed progression
Rapid CNS disease progression during washout period	• Rapid recruitment and enrollment by using a phone line to streamline trial referrals • LAT1 status not required for enrollment
Poor blood–brain barrier penetration of chemotherapy drugs	• Use of LAT1-targeting to both cross the blood–brain barrier and facilitate transport into tumor cells
Limitations of radiographic follow-up	• Alternative biomarkers obtained through CSF biopsy • Quality-of-life survey
Evaluating treatment response of CNS vs. systemic disease	• Routine CNS and systemic imaging
Small sample size	• Expansion to all breast cancer subtypes • Regional academic center collaboration

Initial trial design considerations included allowing for effective treatment of cancer growth in both the brain and body; intrathecal therapies also do not address systemic tumor burden. Systemic administration of treatment for BrM may both mitigate the risk of “re-seeding” compared with CNS local therapies (e.g., radiotherapy, laser interstitial therapy, intrathecal chemotherapy, surgical resection), and has the potential to simultaneously treat systemic disease [48–51]. This is particularly relevant to LMD, given its diffuse nature and hematogenous and/or CSF spread [52]. A blood–brain barrier permeable systemic therapy such as QBS72S is promising due to its potential for treatment of both BrM and systemic disease. Likewise, although initially focused on triple negative breast cancer, our research team rapidly recognized the need to include all breast cancer subtypes given the demand expressed by our Patient Advocates and medical oncology collaborators, bypassing subtype restrictions increasingly common in BrM clinical trials [53]. A key aspect of the design is the inclusion of two concurrent patient cohorts—those with BrM parenchymal disease only and those with LMD, given the distinct survival and symptom profiles. LAT-1 expression status was not required given 1) many patients did not have primary tissue available at the study site, having received care at other facilities, 2) available tissue varied widely (e.g., primary tumor, lymph node, non-CNS metastatic site, brain metastases, LMD cells) with heterogenous progression and duration of treatment, 3) no LAT1 expression grading system previously existed, 4) the level of LAT1 expression had not yet been correlated to QBS72S therapeutic response, and 5) the total time required to acquire tissue and test for LAT1 expression would be prohibitive for rapid enrollment. Finally, given an intervention at the point of LMD is unlikely to be curative, the collection of quality-of-life survey data may support another domain of response determination in this complex patient population.

Patients with BrMs rapidly progress, particularly in LMD where survival is limited to mere months and symptoms can worsen over days to weeks. Recognizing most patients would not have received primary breast cancer treatment at Stanford, we sought to overcome delays in accrual and limit barriers to enrollment by: 1) creating a collaborative network across Stanford, UCSF, and UC Davis for patient referrals while centralizing the administrative burden, 2) establishing a department-supported phone line, with live and immediate access during normal business hours to an administrative facilitator, 3) enabling video visits for pre-screen assessments of patients by Clinical Research Coordinators, 4) rapid and direct communication between the PI and local referring physicians, 5) allowing for post hoc determination of LAT1 target expression, 6) reimbursing patient travel expenses to help defray financial hardship and increase the enrollment and retention of historically underrepresented populations, and 7) tailoring washout periods for radiation and systemic therapy.

Response criteria must effectively measure the impact of the intervention specifically on BrM, considering patients may have no, stable, or progressive systemic disease. Endpoints like PFS and ORR may be dominated by non-CNS disease if not contextualized, and death from neurologic causes can be difficult to determine in a fragmented healthcare system. Additionally, heterogeneity among enrolled patients, prior and subsequent treatment modalities, has hampered previous BrM trials from efficiently gauging treatment effects [5, 54]. We sought to overcome these limitations through data interpretation novelty that included exploratory endpoints including radiographic perfusion protocols, a novel LAT1 grading scale, and the development of novel CSF biomarkers [55]. Pseudoprogression and premature trial termination requires more refined MRI and cytology tools, which we seek to address with perfusion MRI [56], and allowing for a “preliminary disease progression” outcome category. In the LMD cohort, we required a VAD for CSF access to avoid lumbar puncture logistical challenges, allow for testing of intracranial pressure, and determine CSF bioavailability of the drug. To address the question of treatment response and LAT-1 expression, we developed and validated an IHC protocol to assay for relative expression in patient tumor samples. Quantification of LAT1 transporter expression will enable correlation with treatment response to the drug in this and future studies targeting LAT1.

### Novel biomarkers of LMD response assessment are critically needed

Patients with LMD have highly variable disease burden, which cannot be assessed given limitations in the sensitivity and specificity of currently available diagnostic methods (e.g., MRI, cytology) [45, 57, 58]. For example, cytology has a sensitivity of only 50–60% [59–62], with 10% of patients remaining negative despite repeated testing and large CSF volumes [57, 60, 63]. Yet, due to a lack of valid alternatives, clinical trials for LMD have relied upon cytologic analysis of CSF for diagnosis and monitoring [64, 65].

This trial design sought to not only test the efficacy of QBS72S, but also to leverage novel CSF biomarkers to understand disease response, resistance, and toxicity. These tools would allow a more effective and streamlined approach to clinical trials targeting LMD. For example, tumor-associated cell free nucleic acids (cfDNA, cfRNA) have been identified in patient CSF [30, 55, 66–69]. Recent advances in spatial transcriptomics, proteomics, glycoproteomics, single cell RNAseq, and metabolomics, when adapted to CSF, all hold great potential to increase our understanding and treatment of BrM and LMD. We will pursue CSF biomarker development correlated to therapeutic response and resistance. These biomarkers may support more rapid and effective LMD diagnosis and monitoring, overcoming sensitivity limitations of current approaches. The absolute amount of a biomarker of interest (e.g. nucleic acid, protein, cytokine) in CSF is low, but the signal-to-noise ratio may be advantageous in comparison to blood, due to the abundance of genetic material from nonmalignant tissue [70–73]. For example, CSF cfDNA testing has recently been explored for diagnosis of LMD at two large, tertiary medical centers, where it was shown to outperform conventional cytologic assays in sensitivity and accuracy [66]. Therefore, our study’s intentional incorporation of novel CSF-based biomarker development may provide more sensitive measurements of treatment response, resistance, and neurotoxicity

## Conclusions

In summary, there is a large unmet need for effective drugs to treat patients with breast cancer BrM and LMD, with few new compounds currently in development. Adaptive trial design allows for rapid evaluation and enrollment, and post-hoc evaluation of target expression. Novel biomarker tools are needed to determine response to therapy, identify disease progression, and mitigate neurotoxicity in patients with BrM. Blood-brain barrier permeable drugs such as QBS72S hold great promise for patients with currently few treatment or clinical trial options.

## Supplementary Information

Supplementary Material 1.

## Data Availability

No datasets were generated or analysed during the current study.

## Abbreviations

AE: Adverse event
BC: Breast cancer
BrM: Brain metastases
C1D1: Cycle 1, Day 1
CNS: Central nervous system
CSF: Cerebrospinal fluid
CSI: Craniospinal irradiation
DSC: Dynamic susceptibility contrast
DSMC: Data safety monitoring committee
ECG: Electrocardiogram
EIAEDs: Enzyme-inducing antiepileptic drugs
EOI: End of infusion
FDA: Food and drug administration
FLAIR: Fluid Attenuated Inversion Recovery
GBM: Glioblastoma
HER2: Human epidermal growth factor receptor 2
IHC: Immunohistochemical
INR: International normalized ratio
IRB: Institutional review board
IV: Intravenous
kg: Kilograms
KPS: Karnofsky performance status
LAR: Legally authorized representative
LAT1: Large amino transporter type 1
LMD: Leptomeningeal disease
min: Minute
mg: Milligrams
mm: Millimeters
mmHg: Millimeters of mercury
mRANO BM: Modified Response Assessment in Neuro‑Oncology for Brain Metastases
MRI: Magnetic resonance imaging
NSCLC: Non-small cell lung cancer
ORR: Overall response rate
PARP: Poly (ADP-ribose) polymerase
PD: Protocol director
PD-1: Programmed cell death protein 1
PD-L1: Programmed cell death ligand 1
PK: Pharmacokinetic
PI: Principal investigator
q: Approximately
SAE: Serious adverse event
SCI: Stanford cancer institute
SOP: Standard operating procedure
T1 W1: T1-weighted images
T2 W1: T2-weighted images
TNBC: Triple-negative breast cancer
US: United States
VAD: Ventricular access devices
VEGF: Vascular endothelial growth factor

## References

[1] American Brain Tumor Association. (2020). Metastatic Brain Tumors [Brochure]. Chicago, IL: Author. Retrieved 2021, from https://www.abta.org/publications/metastatic-brain-tumors/. In.

[2] Barnholtz-Sloan JS, Sloan AE, Davis FG, Vigneau FD, Lai P, Sawaya RE. Incidence proportions of brain metastases in patients diagnosed (1973 to 2001) in the Metropolitan Detroit Cancer Surveillance System. J Clin Oncol. 2004;22(14):2865–72. 15254054 10.1200/JCO.2004.12.149

[3] Stelzer KJ. Epidemiology and prognosis of brain metastases. Surg Neurol Int. 2013;4(Suppl 4):S192-202. 23717790 10.4103/2152-7806.111296 PMC3656565

[4] Witzel I, Oliveira-Ferrer L, Pantel K, Müller V, Wikman H. Breast cancer brain metastases: biology and new clinical perspectives. Breast Cancer Res. 2016;18:1–9. 26781299 10.1186/s13058-015-0665-1 PMC4717619

[5] Martin AM, Cagney DN, Catalano PJ, Warren LE, Bellon JR, Punglia RS, et al. Brain metastases in newly diagnosed breast cancer: a population-based study. JAMA Oncol. 2017;3(8):1069–77. 28301662 10.1001/jamaoncol.2017.0001 PMC5824221

[6] Sperduto PW, Mesko S, Li J, Cagney D, Aizer A, Lin NU, et al. Beyond an updated graded prognostic assessment (breast GPA): a prognostic index and trends in treatment and survival in breast cancer brain metastases from 1985 to today. International Journal of Radiation Oncology* Biology* Physics. 2020;107(2):334–43. 10.1016/j.ijrobp.2020.01.051 PMC7276246 32084525

[7] Vogelbaum MA, Brown PD, Messersmith H, Brastianos PK, Burri S, Cahill D, et al. Treatment for brain metastases: ASCO-SNO-ASTRO guideline. 2022; 10.1200/JCO.21.02314 34932393

[8] De Azevedo CRAS, Cruz MRS, Chinen LTD, Peres SV, Peterlevitz MA, de Azevedo Pereira AE, et al. Meningeal carcinomatosis in breast cancer: prognostic factors and outcome. J Neurooncol. 2011;104:565–72. 21234642 10.1007/s11060-010-0524-y

[9] Morikawa A, Jordan L, Rozner R, Patil S, Boire A, Pentsova E, et al. Characteristics and outcomes of patients with breast cancer with leptomeningeal metastasis. Clin Breast Cancer. 2017;17(1):23–8. 27569275 10.1016/j.clbc.2016.07.002 PMC5266701

[10] Torrejón D, Oliveira M, Cortes J, Sanchez-Olle G, Gómez P, Bellet M, et al. Implication of breast cancer phenotype for patients with leptomeningeal carcinomatosis. The Breast. 2013;22(1):19–23. 23137566 10.1016/j.breast.2012.10.009

[11] National Institutes of Health. “Leptomeningeal Disease.” NCI Dictionary of Cancer Terms, National Cancer Institute, 2021, www.cancer.gov/publications/dictionaries/cancer-terms/def/leptomeningeal-metastasis. In.

[12] Duelli R, Enerson BE, Gerhart DZ, Drewes LR. Expression of large amino acid transporter LAT1 in rat brain endothelium. J Cereb Blood Flow Metab. 2000;20(11):1557–62. 11083230 10.1097/00004647-200011000-00005

[13] Killian DM, Chikhale PJ. Predominant functional activity of the large, neutral amino acid transporter (LAT1) isoform at the cerebrovasculature. Neurosci Lett. 2001;306(1–2):1–4. 11403943 10.1016/s0304-3940(01)01810-9

[14] El Ansari R, Craze ML, Miligy I, Diez-Rodriguez M, Nolan CC, Ellis IO, et al. The amino acid transporter SLC7A5 confers a poor prognosis in the highly proliferative breast cancer subtypes and is a key therapeutic target in luminal B tumours. Breast Cancer Res. 2018;20:1–17. 29566741 10.1186/s13058-018-0946-6 PMC5863851

[15] Kageyama T, Nakamura M, Matsuo A, Yamasaki Y, Takakura Y, Hashida M, et al. The 4F2hc/LAT1 complex transports L-DOPA across the blood–brain barrier. Brain Res. 2000;879(1–2):115–21. 11011012 10.1016/s0006-8993(00)02758-x

[16] Dickens D, Webb SD, Antonyuk S, Giannoudis A, Owen A, Rädisch S, et al. Transport of gabapentin by LAT1 (SLC7A5). Biochem Pharmacol. 2013;85(11):1672–83. 23567998 10.1016/j.bcp.2013.03.022

[17] Papin-Michault C, Bonnetaud C, Dufour M, Almairac F, Coutts M, Patouraux S, et al. Study of LAT1 expression in brain metastases: towards a better understanding of the results of positron emission tomography using amino acid tracers. PLoS ONE. 2016;11(6): e0157139. 27276226 10.1371/journal.pone.0157139 PMC4898730

[18] Deng J, Chernikova SB, Wang Y, Rodriguez ML, Andersen SJ, Umeh-Garcia MC, et al. A Novel Brain-Permeant Chemotherapeutic Agent for the Treatment of Brain Metastasis in Triple-Negative Breast Cancer. Mol Cancer Ther. 2021;20(11):2110–6. 34635566 10.1158/1535-7163.MCT-21-0140 PMC8571036

[19] Simon R. Optimal two-stage designs for phase II clinical trials. Control Clin Trials. 1989;10(1):1–10. 2702835 10.1016/0197-2456(89)90015-9

[20] ClinicalTrials.gov Identifier: NCT04430842. Dose Escalation Study to Assess the Safety, Tolerability, Pharmacokinetics, and Pharmacodynamics of QBS10072S. Retrieved June, 2024.

[21] Eisenhauer EA, Therasse P, Bogaerts J, Schwartz LH, Sargent D, Ford R, et al. New response evaluation criteria in solid tumours: revised RECIST guideline (version 1.1). European journal of cancer. 2009;45(2):228–47. 10.1016/j.ejca.2008.10.026 19097774

[22] Clopper CJ, Pearson ES. The use of confidence or fiducial limits illustrated in the case of the binomial. Biometrika. 1934;26(4):404–13.

[23] Bland JM, Altman DG. Survival probabilities (the Kaplan-Meier method). BMJ. 1998;317(7172):1572–80. 9836663 10.1136/bmj.317.7172.1572 PMC1114388

[24] Kalbfleisch JD, Prentice RL. The statistical analysis of failure data. IEEE Trans Reliab. 1986;35(1):11–11.

[25] Brookmeyer R, Crowley J. A confidence interval for the median survival time. Biometrics. 1982;29–41.

[26] Rao M. Survival Analysis, Techniques for Censored and Truncated Data. 1998;

[27] Greenwood M. The" errors of sampling" of the survivorship tables. Reports on public health and medical subjects. 1926;

[28] NIH, National Cancer Institute, & Cancer Therapy Evaluation Program. Common terminology criteria for adverse Events (CTCAE) Version 5. Retrieved April, 2021, from https://ctep.cancer.gov/protocolDevelopment/electronic_applications/ctc.htm. In.

[29] Nilapwar SM, Nardelli M, Westerhoff HV, Verma M. Absorption spectroscopy. Methods Enzymol. 2011;500:59–75. 21943892 10.1016/B978-0-12-385118-5.00004-9

[30] Connolly ID, Li Y, Gephart MH, Nagpal S. The, “Liquid Biopsy”: the Role of Circulating DNA and RNA in Central Nervous System Tumors. Curr Neurol Neurosci Rep. 2016;16(3):25. 26838352 10.1007/s11910-016-0629-6 PMC8423147

[31] Takeuchi K, Ogata S, Nakanishi K, Ozeki Y, Hiroi S, Tominaga S, et al. LAT1 expression in non-small-cell lung carcinomas: Analyses by semiquantitative reverse transcription-PCR (237 cases) and immunohistochemistry (295 cases). Lung Cancer. 2010;68(1):58–65. 19559497 10.1016/j.lungcan.2009.05.020

[32] Murthy RK, Loi S, Okines A, Paplomata E, Hamilton E, Hurvitz SA, et al. Tucatinib, Trastuzumab, and Capecitabine for HER2-Positive Metastatic Breast Cancer. N Engl J Med. 2020;382(7):597–609. 31825569 10.1056/NEJMoa1914609

[33] Harbeck N, Ciruelos E, Jerusalem G, Müller V, Niikura N, Viale G, et al. Trastuzumab deruxtecan in HER2-positive advanced breast cancer with or without brain metastases: a phase 3b/4 trial. Nat Med. 2024;30(12):3717–27. 39271844 10.1038/s41591-024-03261-7 PMC11645283

[34] Chen Q, Xiong J, Ma Y, Wei J, Liu C, Zhao Y. Systemic treatments for breast cancer brain metastasis. Front Oncol. 2023;6(12):1086821. 10.3389/fonc.2022.1086821 PMC9853531 36686840

[35] Leone JP, Emblem KE, Weitz M, Gelman RS, Schneider BP, Freedman RA, et al. Phase II trial of carboplatin and bevacizumab in patients with breast cancer brain metastases. Breast Cancer Res. 2020;22(1):131. 33256829 10.1186/s13058-020-01372-w PMC7706261

[36] Bartsch R, Jerzak KJ, Larrouquere L, Müller V, Rhun EL. Pharmacotherapy for leptomeningeal disease in breast cancer. Cancer Treatment Reviews. 2024;122. Available from: https://www.cancertreatmentreviews.com/article/S0305-7372(23)00146-9/fulltext . Cited 2024 Jul 28 10.1016/j.ctrv.2023.102653 38118373

[37] Balinda HU, Kelly WJ, Kaklamani VG, Lathrop KI, Canola MM, Ghamasaee P, et al. Sacituzumab Govitecan in patients with breast cancer brain metastases and recurrent glioblastoma: a phase 0 window-of-opportunity trial. Nat Commun. 2024;15(1):6707. 39112464 10.1038/s41467-024-50558-9 PMC11306739

[38] Li X, Heyer WD. Homologous recombination in DNA repair and DNA damage tolerance. Cell Res. 2008;18(1):99–113. 18166982 10.1038/cr.2008.1 PMC3087377

[39] De Silva IU, McHugh PJ, Clingen PH, Hartley JA. Defining the roles of nucleotide excision repair and recombination in the repair of DNA interstrand cross-links in mammalian cells. Mol Cell Biol. 2000;20(21):7980–90. 11027268 10.1128/mcb.20.21.7980-7990.2000 PMC86408

[40] Ribeiro E, Ganzinelli M, Andreis D, Bertoni R, Giardini R, Fox SB, et al. Triple negative breast cancers have a reduced expression of DNA repair genes. PLoS ONE. 2013;8(6): e66243. 23825533 10.1371/journal.pone.0066243 PMC3692506

[41] Alli E, Sharma VB, Sunderesakumar P, Ford JM. Defective repair of oxidative dna damage in triple-negative breast cancer confers sensitivity to inhibition of poly (ADP-ribose) polymerase. Can Res. 2009;69(8):3589–96. 10.1158/0008-5472.CAN-08-4016 PMC2681413 19351835

[42] Sharma P, Barlow W, Godwin A, Pathak H, Isakova K, Williams D, et al. Impact of homologous recombination deficiency biomarkers on outcomes in patients with triple-negative breast cancer treated with adjuvant doxorubicin and cyclophosphamide (SWOG S9313). Ann Oncol. 2018;29(3):654–60. 29293876 10.1093/annonc/mdx821 PMC5888953

[43] Chopra N, Tovey H, Pearson A, Cutts R, Toms C, Proszek P, et al. Homologous recombination DNA repair deficiency and PARP inhibition activity in primary triple negative breast cancer. Nat Commun. 2020;11(1):2662. 32471999 10.1038/s41467-020-16142-7 PMC7260192

[44] Sharma AE, Corbett K, Soliman H, Sahgal A, Das S, Lim-Fat MJ, et al. Assessment of Phase 3 Randomized Clinical Trials Including Patients With Leptomeningeal Disease: A Systematic Review. JAMA Oncol. 2023;9(4):566–7. 36757707 10.1001/jamaoncol.2022.7364 PMC9912161

[45] Brastianos PK, Lee EQ, Cohen JV, Tolaney SM, Lin NU, Wang N, et al. Single-arm, open-label phase 2 trial of pembrolizumab in patients with leptomeningeal carcinomatosis. Nat Med. 2020;26(8):1280–4. 32483359 10.1038/s41591-020-0918-0

[46] Brastianos PK, Strickland MR, Lee EQ, Wang N, Cohen JV, Chukwueke U, et al. Phase II study of ipilimumab and nivolumab in leptomeningeal carcinomatosis. Nat Commun. 2021;12(1):5954. 34642329 10.1038/s41467-021-25859-y PMC8511104

[47] Yang JT, Wijetunga NA, Pentsova E, Wolden S, Young RJ, Correa D, et al. Randomized phase II trial of proton craniospinal irradiation versus photon involved-field radiotherapy for patients with solid tumor leptomeningeal metastasis. J Clin Oncol. 2022;40(33):3858–67. 35802849 10.1200/JCO.22.01148 PMC9671756

[48] Dawood S, Lei X, Litton JK, Buchholz TA, Hortobagyi GN, Gonzalez-Angulo AM. Incidence of brain metastases as a first site of recurrence among women with triple receptor–negative breast cancer. Cancer. 2012;118(19):4652–9. 22359359 10.1002/cncr.27434 PMC3910255

[49] Pestalozzi BC, Zahrieh D, Price K, Holmberg S, Lindtner J, Collins J, et al. Identifying breast cancer patients at risk for Central Nervous System (CNS) metastases in trials of the International Breast Cancer Study Group (IBCSG). Ann Oncol. 2006;17(6):935–44. 16603601 10.1093/annonc/mdl064

[50] Gainor JF, Ou SHI, Logan J, Borges LF, Shaw AT. The central nervous system as a sanctuary site in ALK-positive non–small-cell lung cancer. J Thorac Oncol. 2013;8(12):1570–3. 24389440 10.1097/JTO.0000000000000029

[51] Wu Y, Zhou L, Lu Y. Intrathecal chemotherapy as a treatment for leptomeningeal metastasis of non-small cell lung cancer: A pooled analysis. Oncol Lett. 2016;12(2):1301–14. 27446430 10.3892/ol.2016.4783 PMC4950629

[52] Franzoi MA, Hortobagyi GN. Leptomeningeal carcinomatosis in patients with breast cancer. Crit Rev Oncol Hematol. 2019;135:85–94. 30819451 10.1016/j.critrevonc.2019.01.020

[53] Lin NU, Prowell T, Tan AR, Kozak M, Rosen O, Amiri-Kordestani L, et al. Modernizing Clinical Trial Eligibility Criteria: Recommendations of the American Society of Clinical Oncology-Friends of Cancer Research Brain Metastases Working Group. JCO. 2017;35(33):3760–73. 10.1200/JCO.2017.74.0761 28968165

[54] Lin NU, Lee EQ, Aoyama H, Barani IJ, Barboriak DP, Baumert BG, et al. Response assessment criteria for brain metastases: proposal from the RANO group. Lancet Oncol. 2015;16(6):e270-278. 26065612 10.1016/S1470-2045(15)70057-4

[55] Li Y, Polyak D, Lamsam L, Connolly ID, Johnson E, Khoeur LK, et al. Comprehensive RNA analysis of CSF reveals a role for CEACAM6 in lung cancer leptomeningeal metastases. NPJ Precis Oncol. 2021;5(1):90. 34625644 10.1038/s41698-021-00228-6 PMC8501028

[56] Kuo F, Ng NN, Nagpal S, Pollom EL, Soltys S, Hayden-Gephart M, et al. DSC Perfusion MRI-Derived Fractional Tumor Burden and Relative CBV Differentiate Tumor Progression and Radiation Necrosis in Brain Metastases Treated with Stereotactic Radiosurgery. AJNR Am J Neuroradiol. 2022;43(5):689–95. 35483909 10.3174/ajnr.A7501 PMC9089266

[57] Straathof CS, de Bruin HG, Dippel DW, Vecht CJ. The diagnostic accuracy of magnetic resonance imaging and cerebrospinal fluid cytology in leptomeningeal metastasis. J Neurol. 1999;246:810–4. 10525979 10.1007/s004150050459

[58] Brastianos PK, Kim AE, Wang N, Lee EQ, Ligibel J, Cohen JV, et al. Palbociclib demonstrates intracranial activity in progressive brain metastases harboring cyclin-dependent kinase pathway alterations. Nature cancer. 2021;2(5):498–502. 35122016 10.1038/s43018-021-00198-5 PMC10644914

[59] Cheng H, Perez-Soler R. Leptomeningeal metastases in non-small-cell lung cancer. Lancet Oncol. 2018;19(1):e43-55. 29304362 10.1016/S1470-2045(17)30689-7

[60] Glantz MJ, Cole BF, Glantz LK, Cobb J, Mills P, Lekos A, et al. Cerebrospinal fluid cytology in patients with cancer: minimizing false‐negative results. Cancer: Interdisciplinary International Journal of the American Cancer Society. 1998;82(4):733–9. 10.1002/(sici)1097-0142(19980215)82:4<733::aid-cncr17>3.0.co;2-z 9477107

[61] Nayar G, Ejikeme T, Chongsathidkiet P, Elsamadicy AA, Blackwell KL, Clarke JM, et al. Leptomeningeal disease: current diagnostic and therapeutic strategies. Oncotarget. 2017;8(42):73312. 29069871 10.18632/oncotarget.20272 PMC5641214

[62] Pan Z, Yang G, He H, Yuan T, Wang Y, Li Y, et al. Leptomeningeal metastasis from solid tumors: clinical features and its diagnostic implication. Sci Rep. 2018;8(1):10445. 29992998 10.1038/s41598-018-28662-w PMC6041294

[63] Grossman SA, Krabak MJ. Leptomeningeal carcinomatosis. Cancer Treat Rev. 1999;25(2):103–19. 10395835 10.1053/ctrv.1999.0119

[64] Phase II trial of ipilimumab and nivolumab in leptomeningeal metastases. ClinicalTrials.gov identifier: NCT02939300. Updated September 7, 2020. Accessed June 17, 2024. https://clinicaltrials.gov/ct2/show/NCT02939300.

[65] Phase II trial of pembrolizumab in central nervous system metastases from multiple histologies. ClinicalTrials.gov identifier: NCT02886585. Updated September 7, 2020. Accessed June 17, 2024. https://clinicaltrials.gov/ct2/show/NCT02886585 9.

[66] White MD, Klein RH, Shaw B, Kim A, Subramanian M, Mora JL, et al. Detection of Leptomeningeal Disease Using Cell-Free DNA From Cerebrospinal Fluid. Vol. 4, JAMA Network Open. 2021. p. e2120040–e2120040. 10.1001/jamanetworkopen.2021.20040 PMC8353541 34369989

[67] Li Y, Liu B, Connolly ID, Kakusa BW, Pan W, Nagpal S, et al. Recurrently Mutated Genes Differ between Leptomeningeal and Solid Lung Cancer Brain Metastases. J Thorac Oncol. 2018;13(7):1022–7. 29604399 10.1016/j.jtho.2018.03.018 PMC8407522

[68] Li Y, Pan W, Connolly ID, Reddy S, Nagpal S, Quake S, et al. Tumor DNA in cerebral spinal fluid reflects clinical course in a patient with melanoma leptomeningeal brain metastases. J Neurooncol. 2016;128(1):93–100. 26961773 10.1007/s11060-016-2081-5 PMC5412509

[69] Pan W, Gu W, Nagpal S, Gephart MH, Quake SR. Brain tumor mutations detected in cerebral spinal fluid. Clin Chem. 2015;61(3):514–22. 25605683 10.1373/clinchem.2014.235457 PMC5412506

[70] Mouliere F, Mair R, Chandrananda D, Marass F, Smith CG, Su J, et al. Detection of cell-free DNA fragmentation and copy number alterations in cerebrospinal fluid from glioma patients. EMBO Mol Med. 2018;10(12): e9323. 30401727 10.15252/emmm.201809323 PMC6284385

[71] Razavi P, Li BT, Brown DN, Jung B, Hubbell E, Shen R, et al. High-intensity sequencing reveals the sources of plasma circulating cell-free DNA variants. Nat Med. 2019;25(12):1928–37. 31768066 10.1038/s41591-019-0652-7 PMC7061455

[72] Alix-Panabières C, Pantel K. Clinical applications of circulating tumor cells and circulating tumor DNA as liquid biopsy. Cancer Discov. 2016;6(5):479–91. 26969689 10.1158/2159-8290.CD-15-1483

[73] Pentsova EI, Shah RH, Tang J, Boire A, You D, Briggs S, et al. Evaluating cancer of the central nervous system through next-generation sequencing of cerebrospinal fluid. J Clin Oncol. 2016;34(20):2404. 27161972 10.1200/JCO.2016.66.6487 PMC4981784

