# Supplementary material for: Adaptive cohort design and LAT1 expression scale: study protocol for a Phase 2a trial of QBS72S in breast cancer brain metastases
Source: BMC Cancer. 2025 Aug 15;25:1316. doi: 10.1186/s12885-025-14282-x (PMC12355770; doi:10.1186/s12885-025-14282-x)
Supplement: Supplementary file 1 — Supplementary Material 1. [file 12885_2025_14282_MOESM1_ESM.docx]

| **Inclusion criteria** | **Exclusion criteria** |
| --- | --- |
| 1. The participant must be 18 years or older. | 1. Participants with any other current, active malignancy unrelated to their primary cancer diagnosis, except for adequately treated basal cell or squamous cell skin cancer or carcinoma in situ. |
| 1. The participant must have a Karnofsky Performance Status (KPS) of 60 or above. | 1. Participants with intolerance to or who have had a severe (Grade 3) allergic or anaphylactic reaction to any of the substances included in the investigational product: sulfobutylether-β-cyclodextrin, melphalan, bendamustine, chlorambucil or any nitrogen mustard chemotherapeutics. |
| 1. One of the following:    1. Cohorts 1 and 2: The participant must have a histologically-confirmed breast cancer primary tumor, either confirmed via primary specimen or via metastasis site, having developed brain metastases (parenchymal or LMD) after a prior cytotoxic chemotherapy regimen.    2. Cohort 3: The participant must have a histologically cancerous primary tumor, either confirmed via primary specimen or via metastasis site, having developed brain metastases (parenchymal or LMD) after a prior cytotoxic chemotherapy regimen.   There is no restriction on prior cycles of systemic therapy for any primary cancer type. Breast cancer is not excluded from Cohort 3. | 1. Participants who currently use or have an anticipated need for a contraindicated medication including live vaccines, natalizumab, nivolumab, ocrelizumab, palifermin, pimecrolimus, tacrolimus, tofacitinib, and EIAEDs, including phenytoin, phenobarbital, carbamazepine, fosphenytoin, primidone, and oxcarbazepine. The washout period for any live vaccine is 30 days prior to enrollment. There is no restriction for seasonal flu vaccines that do not contain live virus, nor any approved COVID vaccine. |
| 1. One of the following:    1. Cohort 1: A participant with breast cancer brain parenchymal tumors must have at least one non-irradiated tumor ≥ 3 mm2 that can be seen on 2 or more separate acquired brain MRI sequences.    2. Cohort 2: A participant with breast cancer primary and LMD must receive either:       1. A brain MRI with contrast that supports the presence of LMD, or       2. A CSF cytology test that is either positive for or suspicious for malignancy. The presence of parenchymal brain metastases does not exclude these participants from Cohort 2.    3. Cohort 3: A participant with LMD from any primary cancer site must receive either:       1. A brain MRI with contrast that supports the presence of LMD, or       2. A CSF cytology test that is either positive for or suspicious for malignancy. The presence of parenchymal brain metastases | 1. Participants who are pregnant or breastfeeding. |
| 1. For corticosteroids and anticonvulsants, the participant must either:    1. not be taking any, or    2. be taking stable or decreasing doses for ≥5 days prior to obtaining the screening Gd-MRI of the brain.   The maximum allowable dose of corticosteroids is 8mg of dexamethasone per day, or the equivalent of another medication as assessed by a Neuro-Oncologist. Escalation of corticosteroids is not allowed ≤14 days prior to C1D1. | 1. Participants who have active medical or psychiatric conditions which, in the opinion of the Principal Investigator or a Sub-Investigator, would compromise or interfere with their ability to participate in the study. |
| 1. The participant must have completed washout from prior therapy before C1D1, as applicable:    1. ≥7 days for Ommaya placement (Cohorts 2+3)    2. ≥14 days for small molecules and non-cytotoxic systemic drugs e.g., PARP inhibitors    3. ≥21 days for checkpoint inhibitors and monoclonal antibodies, e.g., atezolizumab, pembrolizumab, and bevacizumab, and cytotoxic chemotherapy    4. ≥28 days for investigational drugs, radiotherapy, and major surgery. Minor procedures such as tumor biopsy are allowed with written approval from the PI    5. ≥1 dosing cycle for other interventions   All significant toxicities from previous anticancer therapy must have stabilized to a new baseline or have resolved. Participation in long term follow up in another clinical trial is allowed if no procedures will be performed which may interfere with the interpretation of study results. |  |
| 1. The participant must have adequate bone marrow function, including:    1. ANC ≥ 1,500/mm3 or ≥ 1.5 x 109/L    2. Platelets ≥ 100,000/ mm3 or ≥ 100 x 109/L    3. Hemoglobin ≥ 9 g/dL |  |
| 1. The participant must have adequate renal function, including serum creatinine ≤ 1.5 x ULN or estimated creatinine clearance ≥ 50 mL/min. In equivocal cases, a 24-hour urine collection test can be used to estimate the creatinine clearance more accurately. |  |
| 1. The participant must have adequate liver function, including:    1. Total serum bilirubin ≤ 1.5 x ULN unless the participant has documented Gilbert syndrome who must have a total bilirubin < 3.0 mg/dl    2. Aspartate and Alanine aminotransferase (AST and ALT) ≤ 2.5 x ULN; ≤ 5.0 x ULN if there is liver involvement by the tumor |  |
| 1. Any participant physiologically capable of becoming pregnant or getting a partner pregnant must agree to use highly effective contraception during study treatment and for 7 months after study discontinuation. |  |
| 1. Participants or their designated advocates must be willing to and capable of providing informed consent and willing and able to comply with all scheduled visits, treatment plan, laboratory tests, lifestyle considerations, and other study procedures. |  |

**Supplemental Table 1.** Eligibility criteria.
